# PRAME Expression in Mucosal Melanoma of the Head and Neck Region

**DOI:** 10.1097/PAS.0000000000002032

**Published:** 2023-03-13

**Authors:** Costantino Ricci, Maria V. Altavilla, Barbara Corti, Ernesto Pasquini, Livo Presutti, Anna M. Baietti, Luca Amorosa, Tiziana Balbi, Chiara Baldovini, Francesca Ambrosi, Marco Grillini, Antonia D’Errico, Michelangelo Fiorentino, Maria P. Foschini

**Affiliations:** * Pathology Unit; ** ENT Unit, Surgical Department, Maggiore Hospital-AUSL Bologna; # Maxillo-Facial Operative Unit, Bellaria and Maggiore Hospital-AUSL Bologna; † Department of Experimental, Diagnostic, and Specialty Medicine (DIMES), University of Bologna; § Department of Biomedical and Neuromotor Sciences, School of Anatomic Pathology, University of Bologna; ‡ Pathology Unit; ¶ Otolaryngology Unit, Department of Head and Neck Surgery, IRCCS AOUBO; ∥ ENT Unit, Surgical Department; †† Pathology Unit, Department of Biomedical and Neuromotor Sciences (DIBINEM), Bellaria Hospital, University of Bologna, Bologna, Italy

**Keywords:** PRAME, melanocytic, melanoma, mucosa, head & neck, immunohistochemistry

## Abstract

PRAME (PReferentially expressed Antigen in MElanoma), a cancer-testis antigen expressed in normal and neoplastic tissues with several functions, proved to be a useful diagnostic tool in the differential diagnosis between benign and malignant melanocytic lesions. The current study aims to perform PRAME stain on a retrospective case series of mucosal melanocytic tumors of the head and neck region to compare 3 different scores and evaluate the most reliable one in this diagnostic set. Immunohistochemical analysis for PRAME was performed in 54 benign and malignant mucosal melanocytic tumors of the head and neck region collected from 41 patients. The best-performing cutoff of PRAME-positive cells (nuclear stain) to differentiate benign and malignant mucosal melanocytic tumors of the head and neck region is that proposed by Raghavan and colleagues (<60%/≥60% of PRAME-positive cells), with 100% and 77.8% of benign lesions and malignant tumors respectively correctly identified. Applying this score, PRAME stain showed the best results (sensitivity, specificity, accuracy, and positive and negative predictive values) for the diagnosis of head and neck melanocytic tumors. However, a subset of PRAME-negative malignant tumors was identified, especially located in the palatal area (hard and soft palate). Finally, high PRAME expression (≥60%) was associated with specific sites (nasal cavity/nasal septum/turbinates nasopharynx, and the maxillary sinus), nodular histotype, and female sex.

The diagnosis of mucosal melanoma of the head and neck region (MM-H&N) is challenging due to the peculiar anatomic, histologic, and genetic features of these melanocytic tumors.^1^–^6^ MM-H&N are exceedingly rare and even experienced head and neck pathologists struggle to confidently diagnose this entity.^1^–^6^ Besides, in this site it is difficult to benefit from clinical-dermatoscopic correlation (as in the cutaneous counterpart), the samples are often poorly cellular incisional biopsies and/or highly fragmented samples, and some atypical histologic features (epidermal effacement and pagetoid spread of melanocytes in suprabasal layers) are rarely found in the early stage of disease.^5^,^6^ In recent years, several molecular tools, such as fluorescence in situ hybridization (FISH), comparative genomic hybridization, and next-generation sequencing have been implemented to aid in improving the diagnosis of melanocytic tumors.^7^–^10^ Unfortunately, the rarity of MM-H&N and the limited literature data on specific genetic mutations (as *TERT* promoter mutations) and molecular tests (FISH) distinguishing MM-H&N from benign mucosal melanocytic lesions of the head and neck region (MBML-H&N), limits their adoption in this diagnostic field.^1^–^6^,^11^–^15^ The recent introduction of PRAME (PReferentially expressed Antigen in MElanoma) has again shifted interest to using immunohistochemistry for the diagnosis of melanocytic lesions.^16^ PRAME is a cancer-testis antigen isolated in 1997 from autologous T-lymphocytes and direct against the tumor in a patient with metastatic cutaneous melanoma.^17^ In recent years, PRAME was found to be expressed in normal and neoplastic tissues (mainly germ cell tumors of the testis, ovarian cancer, sarcomas, and hematologic tumors), with functions in oncogenesis, immune response evasion, apoptosis, acquisition of metastatic phenotype, and drug resistance.^18^–^24^ PRAME became of great interest for the diagnosis of melanocytic tumors, as it proved to be expressed in melanomas but not in nevi, therefore an immunohistochemical marker of great help in one of the most challenging issues of surgical pathology.^16^,^24^–^41^ However, the majority of previous studies on PRAME did not enroll MM-H&N and the available data on the diagnostic value of PRAME are mainly on skin melanocytic tumors; a few studies only evaluated a limited number of MM-H&N.^16^,^24^–^28^,^40^–^42^ Toyama et al^40^ tested PRAME on a series of differently-sited mucosal melanomas (head and neck region, gastrointestinal, and genitourinary) and obtained promising results. Hovander et al^41^ confirmed these results on a small case series of oral cavity melanomas, and recently Scheurleer et al^42^ found PRAME expression in a cohort of sinonsal melanoma. The present study aims to test and validate PRAME stain as a reliable diagnostic tool in a retrospective case series of MM-H&N, comparing MM-H&N with MBML-H&N.

## MATERIALS AND METHODS

### Case Series (Patients and Specimens) Selection

All MM-H&N and MBML-H&N excised between 2004 and 2022 were retrieved from the database of 3 pathology units: Bellaria Hospital of Bologna (37 cases), Maggiore Hospital of Bologna (9 cases), and IRCCS Azienda Ospedaliero-Universitaria Policlinico di Sant’Orsola, Bologna (8 cases). The patients were selected according to the following inclusion criteria: (1) primary mucosal melanocytic lesion (each case was reviewed to verify its site and exclude metastases and cases more appropriately classifiable as cutaneous, especially for lips); (2) availability of formalin-fixed and paraffin-embedded samples with enough material to perform immunohistochemical analysis; (3) availability of clinical data. By contrast, cases judged as nonprimitive of the mucous membranes, and with no availability of formalin-fixed and paraffin-embedded samples for immunohistochemical analyses and/or clinical data were excluded. For each patient, the following clinical data were recorded: age at first diagnosis and sex. For each MM-H&N sample, the following pathologic features were recorded: type of histologic specimen (excision of the primary tumor, excision of residual tumor/relapse, incisional biopsy), site, histologic subtype, pigmentation, prevalent cytotype, ulceration, bone and/or cartilage infiltration, number of mitoses/mm^2^, lymphovascular invasion, perineural infiltration, and pT stage. For each MBML-H&N, only the following pathologic features were recorded: type of histologic specimen, site, and histologic diagnosis. All cases were reviewed and diagnosed according to the World Health Organization Blue Book on Classification of Head and Neck Tumours (fifth edition, 2022) by a panel of 4 pathologists with specific expertise in melanocytic pathology and/or head and neck pathology (C.R., B.C., T.B., and M.P.F.); all MM-H&N were staged according to the eighth edition of the American Joint Committee on Cancer (AJCC) Cancer Staging Manual.^43^,^44^

### Immunohistochemistry and PRAME Scoring

For each case, a representative slide was selected, and from the corresponding paraffin-embedded tissue block one 3-μm-thick section was cut and stained with Melan A/PRAME (BenchMark ULTRA automated immunostainer; Ventana Medical Systems-Roche Diagnostics; Melan A: clone A103, PRAME: clone EPR20330) according to the previously described and published protocol.^35^,^36^ Cytoplasmatic Melan A stain was identified with the Red detection kit (red cytoplasmatic stain), whereas nuclear PRAME stain with the 3,3′-diaminobenzidine detection kit (brown nuclear stain).^35^,^36^ The double stain Melan A/PRAME was here adopted as it allows to better score PRAME in melanocytic cells and differentiate them from keratinocytes and the background of inflammatory cells.^35^,^36^ In MM-H&N with <76% of PRAME-positive cells (negative according to Lezcan et al^16^) an additional section was stained with single stain for PRAME to evaluate potential discrepancies with Melan A/PRAME.^35^,^36^ To identify the cutoff of PRAME-positive cells more suitable to discriminate MM-H&N and MBML-H&N, 3 different scores utilized in routine practice and/or proposed in the recent literature were tested: (1) score according to Lezcano et al^16^: 0=no expression, 1+=1% to 25%, 2+=26% to 50%, 3+=51% to 75%, 4+= ≥76%, with cases dichotomized in negative (0, 1+, 2+, 3+) and positive (4+); (2) score according to Raghavan et al^30^: 0=no expression—59%, 1+= ≥60%, with cases dichotomized in negative (0) and positive (1+); (3) score according to Santandrea et al^34^: adding the quartile of positive tumor cells (0, 1+, 2+, 3+, 4+ sec. Lezcano et al.) to PRAME expression intensity in tumor cells (0 [no expression], 1+ [weak], 2+ [moderate], 3+ [strong]), with cases dichotomized in negative (<5) and positive (≥5).

### Statistical Analysis

For each score sensitivity (SE), specificity (SP), positive predictive value (PPV), negative predictive value (NPV), and accuracy (AC) were evaluated. The score with the highest values of these parameters has been chosen to dichotomize the cases (low and high expression of PRAME) and analyze the association with the other dichotomous/categorical clinicopathologic features using the χ^2^ test. The statistical tests were performed using the IBM SPSS software, with a *P*-value <0.05 (2-sided) indicating statistical significance.

### Ethical Approval

The study has been approved by the Review Board of the Area Vasta Emilia Centro-AVEC (protocol n. 03-2022-OSS-AUSLBO).

## RESULTS

### Case Series (Patients and Specimens)

**FIGURE 1:**
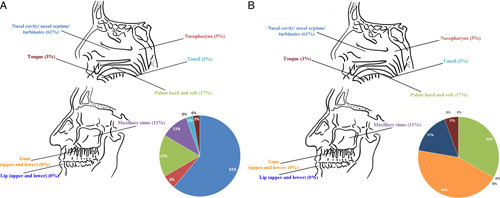
Graphical representation of MM-H&N (A) and MBML-H&N (B) in our case series.

A total of 54 histologic samples from 41 patients were collected. Samples consisted of 37 (68.5%) excisions of the primary tumor, 10 (18.5%) excisions of residual tumor/relapse, and 7 (8.5%) incisional biopsies. Twenty-five (61%) patients were females and 16 (39%) were males; the age at diagnosis ranged from 10 to 96 years (median value: 60 y). Clinicopathologic features of the case series are summarized in Tables 1 and 2. A graphical representation of MM-H&N and MBML-H&N sites is provided in Figure 1.

**TABLE 1:** Clinicopathologic Features of the Case Series

	MM-H&N and MBML-H&N, n (%)	MM-H&N, n (%)	MBML-H&N, n (%)
	Patients (N=41)	Patients (N=23)	Patients (N=18)
Clinical features			
Age, median value (range)	60 (10-96)	69.9 (29-96)	47.4 (10-79)
Sex			
Male	16 (39)	9 (39.1)	7 (38.9)
Female	25 (61)	14 (60.9)	11 (61.1)
	Histologic samples (N=54)	Histologic samples (N=36)	Histologic samples (N=18)
Pathologic features			
Site			
Nasal cavity/nasal septum/turbinates	22 (40.7)	22 (61.1)	0 (0)
Nasopharynx	2 (3.7)	2 (5.6)	0 (0)
Palate (hard and soft)	12 (22.2)	6 (16.7)	6 (33.3)
Maxillary sinus	4 (7.4)	4 (11)	0 (0)
Tonsil	1 (1.9)	1 (2.8)	0 (0)
Gum (upper and lower)	8 (14.8)	0 (0)	8 (44.4)
Lip (upper and lower)	3 (5.6)	0 (0)	3 (16.7)
Tongue	2 (3.7)	1 (2.8)	1 (5.6)
Type of histologic specimen			
Excision of the primary tumor	37 (68.5)	24 (66.7)	13 (72.2)
Excision of residual tumor/relapse	10 (18.5)	10 (27.8)	0 (0)
Incisional biopsy	7 (13)	2 (5.5)	5 (27.8)

MM-H&N: mucosal melanoma of the head and neck region; MBML-H&N: mucosal benign melanocytic lesions of the head andneck region.

**TABLE 2:** Pathologic Features of MM-H&N and Histologic Diagnosis of MBML-H&N

	MM-H&N, n (%)	
	Patients (N=23)	Samples (N=36)
Histologic subtype		
Nodular	14 (60.9)	16 (44.4)
Mucosal lentiginous	9 (39.1)	20 (55.6)
Pigmentation		
Yes	15 (65.2)	9 (25)
No	8 (34.8)	27 (75)
Prevalent cytotype		
Epithelioid	14 (60.9)	20 (55.6)
Fused	5 (21.7)	11 (30.5)
Mixed	4 (17.4)	5 (13.9)
Ulceration		
Yes	18 (78.3)	7 (19.4)
No	5 (21.7)	29 (80.6)
Bone and/or cartilage infiltration		
Yes	6 (26.1)	7 (19.4)
No	17 (73.9)	29 (80.6)
Mitoses/mm², median value (range)	—	5 (1-13)
LVI		
Yes	10 (43.5)	23 (63.9)
No	13 (56.5)	13 (36.1)
PNI		
Yes	2 (8.7)	34 (94.4)
No	21 (91.3)	2 (5.6)
pT stage		
pT3	18 (78.3)	29 (80.6)
pT4a	4 (17.4)	6 (16.7)
pT4b	1 (4.3)	1 (2.8)
	MBML-H&N, n (%)	
	Patients and samples (N=18)	
Histologic diagnosis		
Melanotic macula	10 (55.6)	
Blue nevus	6 (33.3)	
Common nevus	2 (11.1)	

Herein, we reported values for patients and samples because, for the cases of MM-H&N with multiple samples, a global (column: patients) and a single-sample (column: samples) assessment of the histologic features was performed.

LVI indicates lymphovascular invasion; PNI, perineural infiltration.

### MBML-H&N (Clinicopathologic Features and PRAME Expression)

**FIGURE 2:**
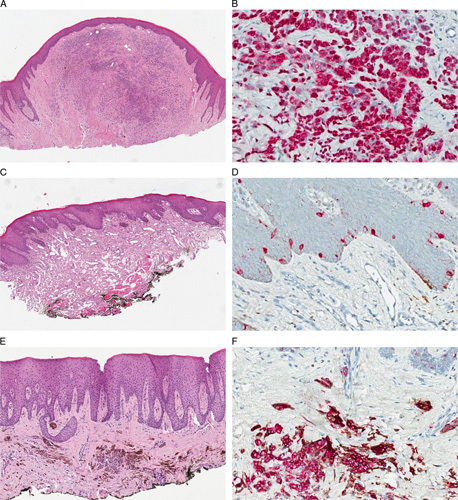
MBML-H&N: mucosal benign melanocytic lesions of the head and neck region; PRAME expression in MBML-H&N. Three cases of histologically straightforward MBML-H&N: common nevus (A: hematoxylin and eosin), melanotic macula (C: hematoxylin and eosin), and blue nevus (E: hematoxylin and eosin), respectively. Immunohistochemistry for Melan A/PRAME shows no or focal PRAME expression in the nuclei of melanocytes (B, D, F: PRAME).

Eighteen histologic samples from 18 patients were collected: 13 (72.2%) excisions of the primary lesion and 5 (27.8%) incisional biopsies. Eleven (61.1%) patients were females and 7 (38.9%) males; the age at diagnosis ranged from 10 to 79 years (median value: 47.4 y). The most represented sites were gum (8, 44.4%) and palate (6, 33.3%). The cases were diagnosed as follows: 10 (55.6%) melanotic macules, 6 (33.3%) blue nevi, and 2 (11.1%) common nevi. All MBML-H&N resulted negative for nuclear PRAME expression according to the 3 tested scores. Thirteen (72.2%) cases were completely negative, 5 (27.8%) showed focal and weak immunoreactivity in a low percentage of cells (5% to 15%) with a random distribution and no intralesional intensity variation (defined as at least 2 adjacent high-power fields with >75% stain in a tumor with an overall uniform stain <75%) (Fig. 2).^27^

### MM-H&N (Clinicopathologic Features and PRAME Expression)

**FIGURE 3:**
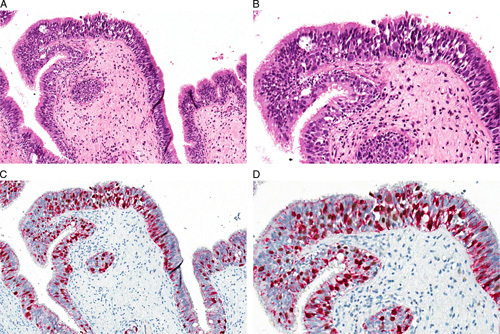
PRAME expression in a case of mucosal lentiginous MM-H&N. The histologic examination shows the intraepithelial component (A, B: hematoxylin and eosin) nicely highlighted by nuclear PRAME stain (brown) (C: hematoxylin and eosin; D: PRAME), and so being potentially useful for the assessment of mucosal resection margins.

**FIGURE 4:**
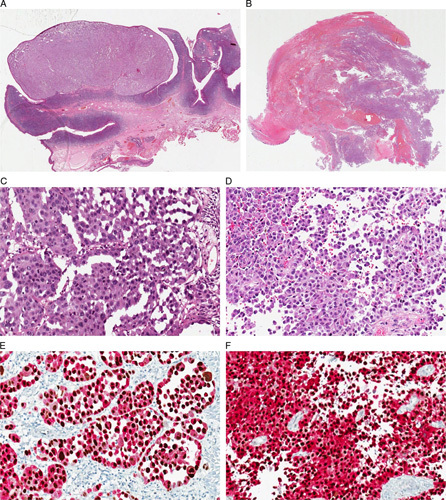
Two cases of nodular MM-H&N. Large and polypoid lesion (A: hematoxylin and eosin), with a nodular growth of atypical melanocytes (C: hematoxylin and eosin) diffusely positive for PRAME (brown nuclei) and Melan A (red cytoplasm) (E: PRAME). Highly fragmented sample of an ulcerated MM-H&N (B: hematoxylin and eosin) with a diffuse growth of atypical melanocytes (D: hematoxylin and eosin) diffusely positive for PRAME (brown nuclei) and Melan A (red cytoplasm) (F: PRAME).

**FIGURE 5:**
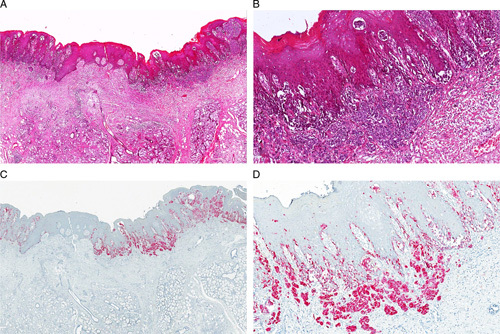
MBML-H&N: mucosal benign melanocytic lesions of the head and neck region; Negative PRAME expression in a case of mucosal lentiginous MM-H&N of the palate. The histologic examination shows a contiguous proliferation of atypical non-nested junctional melanocytes and irregular/dyschoesive nests at the dermoepidermal junction and in the superficial portion of the lamina propria, with marked epidermal effacement and diffuse pagetoid spread (A, C: hematoxylin and eosin). Double stain for Melan A/PRAME shows as the entire melanocytic population is PRAME-negative, with scattered positive fibroblasts as a positive control (B, D: hematoxylin and eosin).

Thirty-six histologic samples from 23 patients were collected: 24 (66.72%) excisions of the primary tumor, 10 (27.8%) excisions of residual tumor/relapse, and 2 (5.5%) incisional biopsies, both followed by the surgical excision of the primary tumor. Fourteen (60.9%) patients were females and 9 (39.1%) males; the age at diagnosis ranged from 29 to 96 years (median value: 69.9 y). The most represented sites were the nasal cavity/nasal septum/turbinates (22, 40.7%) and palate (6, 16.7%). The most frequent histologic subtype was the nodular one (14, 60.9%), with a high percentage of cases showing ulceration (18, 78.3%) and prevalent epithelioid cytology (14, 60.9%). According to the eighth edition of the AJCC Cancer Staging Manual, the pT stages included 18 (78.3%) pT3, 4 (17.4%) pT4a, and 1 (4.3%) pT4b. MM-H&N were characterized by a diffuse (≥60% of neoplastic cells) nuclear expression of PRAME in 28 (77.8%) cases, and with a moderate/intense expression (intensity 2+ and 3+) in 20 (55.6%) (Figs. 3, 4). A single stain for PRAME was additionally performed in all cases of MM-H&N with <76% of PRAME-positive cells (negative according to Lezcano et al^16^) and the results were superimposable to those observed with double stain for Melan A/PRAME.^35^ The two incisional biopsies of MM-H&N were both positive only adopting the score of Raghavan et al^30^ (PRAME-positive cells: 70% and 65%, respectively). Noteworthy, both patients showed higher expression of PRAME (percentage of positive cells and/or intensity) in the subsequent surgical excisions resulting positive with all 3 tested scores.^16^,^30^,^34^ Two cases of MM-H&N were completely negative (0% of positive cells) for PRAME (Fig. 5), and both were located in the palate. In one of them, the negativity was probably related to the quality of the histologic sample (extensive necrosis and poor fixation).

### Comparison of PRAME Scores for the Diagnosis of MBML-H&N and MM-H&N

**FIGURE 6:**
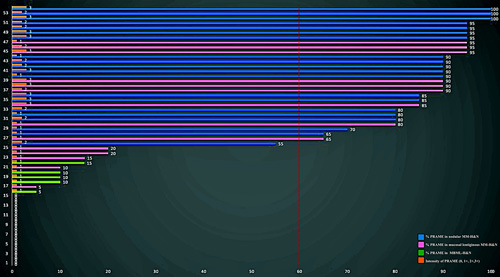
Graphical representation of PRAME expression (% of positive cells and intensity) in MBML-H&N and MM-H&N. Each case corresponds to 2 bars, one for the % of PRAME-positive cells (green: MBML-H&N, pink: mucosal lentiginous MM-H&N, blue: nodular MM-H&N) and one for the intensity (orange bars). Fifteen cases showed 0% of PRAME-positive cells (13 MBML-H&N and 2 mucosal lentiginous MM-H&N; see the Results section and Fig. 5 for more explanations). Notably, the majority of MM-H&N below the cutoff of 60% of PRAME-positive cells (red dotted line) are mucosal lentiginous (only 1 case was nodular); all MBML-H&N show % of PRAME-positive cells below this threshold.

All the tested scores^16^,^30^,^34^ showed SP and PPV of 100%, since no MBML-H&N turned out positive with any score. The score of Raghavan et al^30^ exhibited the highest values of SE, NPV, and AC: 77.8%, 69.2%, and 81.5%, respectively. The values of PRAME (percentage of positive cells and intensity) are summarized in Supplemental Digital Content 1 (http://links.lww.com/PAS/B522). The overview of PRAME results in MM-H&N and MBML-H&N, and the comparison between the 3 different scores are provided in Table 3; a graphical representation of MM-H&N and MBML-H&N cases distribution according to PRAME stain is shown in Figure 6.

**TABLE 3:** The Overview of PRAME Results and the Comparison Between the 3 Different Scores for the Diagnosis of MBML-H&N and MM-H&N

	MBML-H&N, n/N (%)				MM-H&N, n/N (%)			
	Common nevus	Blue nevus	Melanotic macula	All	Mucosal lentiginous	Nodular	All	
Lezcano et al^16^								
0, 1+, 2+, 3+	2/2 (100)	6/6 (100)	10/10 (100)	18/18 (100)	8/16 (50)	3/20 (15)	11/36 (30.6)	SE: 69.4% SP: 100% PPV: 100% NPV: 62% AC: 79.6%
4+	0 (0)	0 (0)	0 (0)	0 (0)	8/16 (50)	17/20 (85)	25/36 (69.4)	
Raghavan et al^30^								
0	2/2 (100)	6/6 (100)	10/10 (100)	18/18 (100)	7/16 (43.7)	1/20 (5)	8/36 (22.2)	SE: 77.8% SP: 100% PPV: 100% NPV: 69.2% AC: 85.2%
1+	0 (0)	0 (0)	0 (0)	0 (0)	9/16 (56.3)	19/20 (95)	28/36 (77.8)	
Santandrea et al^34^								
<5	2/2 (100)	6/6 (100)	10/10 (100)	18/18 (100)	8/16 (50)	2/20 (10)	10/36 (27.8)	SE: 72.2% SP: 100% PPV: 100% NPV: 64.3% AC: 81.5%
≥5	0 (0)	0 (0)	0 (0)	0 (0)	8/16 (50)	18/20 (90)	26/36 (72.2)	

MM-H&N: mucosal melanoma of the head and neck region; MBML-H&N: mucosal benign melanocytic lesions of the head andneck region.

### Association Between PRAME Expression and Clinicopathologic Features

Based on the obtained results, the cutoff of 60% of PRAME-positive cells (nuclear stain) was chosen to dichotomize MM-H&N cases in high (≥60%) and low (<60%) expression, and analyze the association of PRAME expression with the other dichotomous/categorical clinicopathologic features. Accordingly, high expression of PRAME was associated with female sex (21/23 [91.3%] vs. 7/13 [53.8%] in male sex, *P*=0.016) and nodular histologic subtype (19/20 [95%] vs. 9/16 [56.8%] in mucosal lentiginous one, *P*=0.005). Notably, although the mucosal lentiginous histotype showed lower rates of positivity, PRAME highlighted the intraepithelial component/growth, so being potentially useful for the appropriate evaluation of the mucosal resection margins (Fig. 3). High expression of PRAME was also significantly associated with specific anatomic sites (*P*<0.001), as follows: MM-H&N of the nasal cavity/nasal septum/turbinates showed high expression of PRAME in 20/22 (90.9%) cases, whereas MM-H&N of the palate in 0/6 (0%). Albeit affected by much fewer cases, all the cases of the nasopharynx (2/2, 100%) and the maxillary sinus (4/4, 100%) exhibited high expression of PRAME. No statistically significant association was found between high expression of PRAME and the other clinicopathologic features (type of histologic specimen, pigmentation, prevalent cytotype, ulceration, bone and/or cartilage infiltration, lymphovascular invasion, perineural infiltration, and pT stage) (Table 4).

**TABLE 4:** The Association Between Clinicopathologic Features and PRAME Expression (Low: <60%, High ≥60) in MM-H&N

	MM-H&N, samples (N=36), n (%)			
	Low expression of PRAME (<60)		High expression of PRAME (≥60)	*P*
Type of histologic specimen				
Excision of the primary tumor	7 (19.4)	17 (47.2)		0.349
Excision of residual tumor/relapse	1 (2.8)	9 (25)		
Incisional biopsy	0 (0)	2 (5.6)		
Sex				
Male	6 (16.7)	7 (19.4)		0.009
Female	2 (5.6)	21 (58.3)		
Site				
Nasal cavity/nasal septum/turbinates	2 (5.6)	20 (55.6)		<0.001
Nasopharynx	0 (0)	2 (5.6)		
Palate (hard and soft)	6 (16.7)	0 (0)		
Maxillary sinus	0 (0)	4 (14.3)		
Tonsil	0 (0)	1 (2.8)		
Tongue	0 (0)	1 (2.8)		
Histologic subtype				
Mucosal lentiginous	7 (19.4)	9 (25)		0.005
Nodular	1 (2.8)	19 (52.8)		
Pigmentation				
No	2 (5.6)	7 (19.4)		1.000
Yes	6 (16.7)	21 (58.3)		
Prevalent cytotype				
Epithelioid	4 (14.3)	16 (44.4)		0.890
Fused	3 (8.3)	8 (22.2)		
Mixed	1 (2.8)	4 (14.3)		
Ulceration				
No	2 (5.6)	5 (13.9)		0.497
Yes	6 (16.7)	23 (63.9)		
Bone and/or cartilage infiltration				
No	0 (0)	7 (19.4)		0.115
Yes	8 (22.2)	21 (58.3)		
LVI				
No	5 (13.9)	18 (50)		0.926
Yes	3 (8.3)	10 (27.8)		
PNI				
No	7 (19.4)	27 (75)		0.331
Yes	1 (2.8)	1 (2.8)		
pT stage				
pT3	5 (13.9)	24 (66.7)		0.184
pT4a	3 (8.3)	3 (8.3)		
pT4b	0 (0)	1 (2.8)		

LVI indicates lymphovascular invasion; PNI, perineural infiltration.

## DISCUSSION

After the introduction of immunohistochemistry for PRAME in the diagnosis of melanocytic lesions, many researchers focused on its application in several diagnostic scenarios (Spitz lesions, lentigo maligna, acral lesions, etc.) with encouraging but also partially discording results.^16^,^24^–^41^ First, it is questionable whether PRAME stain could be interchangeable and therefore completely replace biomolecular tests for the assessment of “difficult-to-classify tumors,” even if it showed high correlation (about 90%) with cytogenetic tests (FISH and single-nucleotide polymorphism array) in case series enrolling a large spectrum of challenging melanocytic tumors.^26^–^30^ Furthermore, despite the most diffuse and applied immunohistochemical score of PRAME is that proposed by Lezcano and colleagues, several authors found that alternative scores (different cutoffs and/or a combination of a qualitative and quantitative assessment) are more performing for the assessment of specific histotypes of melanocytic lesions.^16^,^24^–^42^ As a result, at the state of the art, it is not known whether the PRAME score should be one in all cases, or if different scores depending on the analyzed tumor are more appropriate.^16^,^24^–^42^ To the best of our knowledge, this is the first study testing PRAME and comparing different scores for the appropriate diagnosis of MM-H&N and MBML-H&N. A heterogenous case series of MBML-H&N, including nodular and mucosal lentiginous mucosal melanoma, different histologic subtypes of MBML-H&N (melanotic macula, blue nevus, and common nevus), and covering a large spectrum of sites (nasal cavity/nasal septum/turbinates, palate, nasopharynx, etc.) constituted the basis of the present study. The most relevant data emerging is that all MBML-H&N are negative for PRAME with all 3 tested scores.^16^,^30^,^34^ Namely, the majority of MBML-H&N showed no PRAME immunostaining, whereas a minority of cases showed rare positive cells only, scattered through the lesion without intralesional intensity variation and hotspots.^27^ As a result, SP and PPV were 100% (0 false-positive cases) with all 3 tested scores.^16^,^30^,^34^ This result can be relevant in a diagnostic scenario where each false-positive case risks being subjected to aggressive therapies impacting on the quality of life.^1^–^6^ According to the data here shown, the most performing score for differentiating MM-H&N from MBML-H&N (SE: 77.8%; NPV: 69.2%; AC: 81.5%) was that proposed by Raghavan et al.^30^ They analyzed a heterogenous case series of melanocytic tumors with intermediate histopathologic or spitzoid features, and found that the best threshold to differentiate benign from malignant tumors was 60% of PRAME-positive cells.^30^ According to these authors, the loss of SE obtained with the other scores would impair the utility of PRAME.^16^,^30^,^34^ This finding highlights as scores alternative to that of Lezcano and colleagues may be more effective for specific categories of melanocytic lesions, as previously found in other scenarios (metastatic melanoma, nodal nevi vs. nodal metastatic melanoma, nevus-associated melanoma, conjunctival, acral, and nail unit melanocytic lesions).^31^–^39^ Additional aspects emerging from the present data, that should be analyzed before the adoption of PRAME for the routine diagnosis of lesions are: (1) different PRAME results according to the site; (2) PRAME results in the incisional biopsies. The data here shown highlight that PRAME must always be analyzed according to the topography of the melanocytic tumor and that a negative stain should be carefully interpreted in a palatal lesion morphologically suggestive for MM-H&N. In the present case series, the MM-H&N incisional biopsies were two only, but both positive only adopting the cutoff of Raghavan et al^30^ (Supplemental Digital Content 1, http://links.lww.com/PAS/B522). By contrast, the subsequent surgical excisions of both patients turned out positive with all 3 scores. This finding suggests that, due to the well-known heterogeneity of PRAME expression and the little amount of tissue often obtained with incisional biopsies, PRAME could be more appropriately scored in the incisional biopsies by adopting a lower threshold of PRAME-positive cells. Notably, all 5 incisional biopsies of MBML-H&N resulted negative with all 3 tested scores. Based on the present findings, it is proposed that positive PRAME stain strongly encourages a diagnosis of MM-H&N, since we did not find MBML-H&N positive for PRAME. By contrast, if the histopathologic features are strongly suggestive for MM-H&N but PRAME stain is negative, as it happens in a not negligible number of MM-H&N and especially in specific sites (palate), the possibility of malignancy should not be discarded. Finally, if the histopathologic features are supportive for MBML-H&N, PRAME stain is expected to be negative and a positive result should lead to reconsidering the possibility of malignancy. An additional benefit of PRAME could be the assessment of the mucosal resection margins, especially in cases of lentiginous MM-H&N. This latter, although with a lower positivity (9/16, 56.8%) compared with the nodular one (19/20, 95%), exhibited an intraepithelial component nicely depicted by Melan A/PRAME (Fig. 3). This scenario mirrors what found by Gradecki et al^32^ in skin lentigo maligna, where the authors suggested that the double stain Melan A/PRAME is helpful. Finally, the present findings indicate that high PRAME expression (≥60%) is significantly associated with specific sites, nodular histotype, and female sex (Table 4). Although the involved pathogenetic mechanisms for justifying these associations have been poorly investigated and are beyond the scope of this article, they should be kept in mind to avoid misdiagnoses. High nuclear PRAME expression (≥60%) was preferentially detected in MM-H&N of the nasal cavity/nasal septum/turbinates (20/22, 90.9%), nasopharynx (2/2, 100%) and maxillary sinus (4/4, 100%) rather than palate (0/6, 0%). Scheurleer et al^42^ found PRAME expression in all tested cases (23/23, 100%) of MM-H&N of the sinonasal region, but they did not test MM-H&N of other sites (palate, nasopharynx, etc.). In the study of Hovander et al,^41^ 6/7 (86%) MM-H&N of the oral cavity/palate resulted positive for PRAME; nevertheless, the authors did not report the criteria used for PRAME assessment (score and cutoff) and so it is not possible to compare their results with the present ones. Toyama et al^40^ did not find statistically significant associations between PRAME expression and tumor site in mucosal melanomas. However, Toyama et al’s^40^ study differs from the present one for the tested case series (sinonasal vs. nonsinonasal sites), PRAME assessment (*H*-score), and adopted statistical test (comparison of the mean values with a *t* test calculator). A large amount of evidences emerging from recent literature data suggests that, although MM-H&N are grouped as such unique subgroup for classification purposes, they should be more appropriately considered as a heterogenous family of tumors, with marked differences from clinical, histologic, immunohistochemical, molecular, and pathogenetic sides.^1^–^6^,^11^,^14^,^40^ According with this theory and with a classification of melanomas progressively based on their molecular profile, MM-H&N of different sites exhibit divergent mutational landscapes and should be probably designed and classified as different lesions.^14^,^15^ Öztürk Sari et al^15^ found that 91% of sinonasal but only 9% of oral MM-H&N harbored mutations in the tested genes (*BRAF*, *NRAS*, *KIT*, *TERT*, *GNAQ*/*GNA11*); besides, *NRAS* and *TERT* promoter mutation were significantly higher in sinonasal than in oral location. More recently, Chłopek et al^14^ analyzed a large case series of sinonasal melanomas and found molecular divergences between paranasal sinuses (10/14 [71%] *BRAF*/*RAS* mutants) and nasal (26/64 [41%] *BRAF*/*RAS* mutants) cases. The present results support the existence of differences among MM-H&N arising in different mucosal sites and suggest that PRAME, as a pivot molecule in the biology of melanoma, could be differently involved and expressed in distinct sites.^14^–^17^ The high expression of PRAME primarily found in nodular (19/20, 95%) rather than mucosal lentiginous histotype (9/16, 56.8%) reflects what previously found in the skin for other subtypes of melanocytic lesions (acral melanocytic lesions, spitzoid lesions, lentigo maligna, nevus-associated melanomas, etc.).^16^,^26^–^42^ It could be explained by assuming that histologic differences reflects clinical, immunohistochemical, but primarily molecular differences, thus potentially justifying a selective involvement of PRAME in their pathogenesis.^1^–^6^,^11^,^14^,^16^,^26^,^43^ The higher PRAME expression in the female sex (21/23, 91.3%) rather than in male one (7/13, 53.8%) is more difficult to justify. It is possible that the peculiar role of sex hormones (androgen and estrogen) in the development of melanomas, as well as the complex intracellular mechanisms regulated by these 2 molecules, could explain the different PRAME expression observed in females and males.^45^,^46^ To conclude, the data here obtained indicate that PRAME is a useful tool for the appropriate diagnosis of MBML-H&N and MM-H&N. The most reliable score to differentiate MM-H&N and MBML-H&N is that proposed by Raghavan and colleagues (< 60% vs. ≥60% of PRAME-positive cells), but a subgroup of specifically sited MM-H&N (palate) can be PRAME-negative (although this assumption is based on a low number of cases and future studies are needed to verify this finding). High PRAME expression (≥60%) was found in association with specific mucosal sites, nodular histotype, and female sex, suggesting a distinct involvement of this molecule in the pathogenesis of these subgroups of tumors.

## Supplementary Material

SUPPLEMENTARY MATERIAL
