# Supplementary material for: PRAME Expression in Mucosal Melanoma of the Head and Neck Region
Source: Am J Surg Pathol. 2023 Mar 13;47(5):599–610. doi: 10.1097/PAS.0000000000002032 (PMC10101133; doi:10.1097/PAS.0000000000002032)
Supplement: SUPPLEMENTARY MATERIAL [file pas-47-599-s001.docx]

| Patient number | Case number | Diagnosis | Site | Type of  histological sample | PRAME (% of positive cells) | Lezcano C  et al^16^ | Raghavan SS  et al^30^ | PRAME (intensity of stain)^34^ | Santandrea G  et al^34^ |
| --- | --- | --- | --- | --- | --- | --- | --- | --- | --- |
| 1 | 1 | ML | NC/NS/T | EPT | 95 | + | + | 3 | + |
|  | 2 |  | NC/NS/T | ERT/R | 90 | + | + | 2 | + |
| 2 | 3 | N | NC/NS/T | IB | 70 | - | + | 1 | - |
|  | 4 |  | NC/NS/T | EPT | 90 | + | + | 1 | + |
| 3 | 5 | ML | NC/NS/T | EPT | 85 | + | + | 3 | + |
| 4 | 6 | N | NC/NS/T | EPT | 90 | + | + | 3 | + |
| 5 | 7 | N | NC/NS/T | EPT | 80 | + | + | 2 | + |
|  | 8 |  | NC/NS/T | ERT/R | 85 | + | + | 3 | + |
|  | 9 |  | Nasopharynx | ERT/R | 100 | + | + | 3 | + |
|  | 10 |  | Nasopharynx | ERT/R | 100 | + | + | 2 | + |
| 6 | 11 | N | NC/NS/T | EPT | 100 | + | + | 3 | + |
| 7 | 12 | ML | Maxillary sinus | EPT | 80 | + | + | 1 | + |
| 8 | 13 | ML | NC/NS/T | EPT | 20 | - | - | 1 | - |
| 9 | 14 | N | NC/NS/T | EPT | 80 | + | + | 1 | + |
| 10 | 15 | N | NC/NS/T | EPT | 55 | - | - | 2 | + |
| 11 | 16 | ML | Palate | EPT | 0 | - | - | 0 | - |
| 12 | 17 | N | Tonsil | EPT | 90 | + | + | 2 | + |
| 13 | 18 | ML | NC/NS/T | IB | 65 | - | + | 1 | - |
|  | 19 |  | NC/NS/T | EPT | 90 | + | + | 3 | + |
|  | 20 |  | NC/NS/T | ERT/R | 95 | + | + | 2 | + |
|  | 21 |  | NC/NS/T | ERT/R | 90 | + | + | 3 | + |
|  | 22 |  | Maxillary sinus | ERT/R | 95 | + | + | 1 | + |
| 14 | 23 | N | NC/NS/T | EPT | 90 | + | + | 2 | + |
|  | 24 |  | NC/NS/T | ERT/R | 90 | + | + | 1 | + |
| 15 | 25 | N | NC/NS/T | EPT | 95 | + | + | 3 | + |
| 16 | 26 | N | NC/NS/T | EPT | 95 | + | + | 3 | + |
| 17 | 27 | N | Maxillary sinus | EPT | 95 | + | + | 2 | + |
|  | 28 |  | Maxillary sinus | ERT/R | 80 | + | + | 2 | + |
| 18 | 29 | ML | Palate | EPT | 5 | - | - | 1 | - |
| 19 | 30 | N | NC/NS/T | EPT | 95 | + | + | 2 | + |
| 20 | 31 | N | NC/NS/T | EPT | 65 | - | + | 1 | - |
| 21 | 32 | ML | Palate | EPT | 20 | - | - | 1 | - |
|  | 33 |  | Palate | EPT | 0 | - | - | 0 | - |
| 22 | 34 | ML | Palate | EPT | 10 | - | - | 1 | - |
|  | 35 |  | Palate | ERT/R | 15 | - | - | 1 | - |
| 23 | 36 | N | Tongue | EPT | 85 | + | + | 3 | + |
| 24 | 37 | MM | Gum | IB | 0 | - | - | 0 | - |
| 25 | 38 | BN | Palate | EPT | 0 | - | - | 0 | - |
| 26 | 39 | CM | Lip | EPT | 0 | - | - | 0 | - |
| 27 | 40 | MM | Lip | IB | 10 | - | - | 1 | - |
| 28 | 41 | CM | Gum | EPT | 0 | - | - | 0 | - |
| 29 | 42 | BN | Gum | EPT | 15 | - | - | 1 | - |
| 30 | 43 | MM | Palate | EPT | 0 | - | - | 0 | - |
| 31 | 44 | MM | Gum | IB | 0 | - | - | 0 | - |
| 32 | 45 | MM | Gum | IB | 0 | - | - | 0 | - |
| 33 | 46 | MM | Palate | EPT | 0 | - | - | 0 | - |
| 34 | 47 | MM | Gum | EPT | 0 | - | - | 0 | - |
| 35 | 48 | MM | Lip | EPT | 10 | - | - | 1 | - |
| 36 | 49 | MM | Gum | EPT | 0 | - | - | 0 | - |
| 37 | 50 | MM | Palate | EPT | 5 | - | - | 1 | - |
| 38 | 51 | BN | Gum | IB | 10 | - | - | 1 | - |
| 39 | 52 | BN | Palate | EPT | 0 | - | - | 0 | - |
| 40 | 53 | BN | Tongue | EPT | 0 | - | - | 0 | - |
| 41 | 54 | BN | Palate | EPT | 0 | - | - | 0 | - |

**Supplemental Digital Content Legend**

**Supplemental Digital Content 1.**

ML: mucosal lentiginous; N: nodular; MM: melanotic macula; CN: common nevus; BN: blue nevus; NC/NS/T: nasal cavity/nasal septum/turbinates; EPT: excision of the primary tumor; ERT/R: excision of residual tumor/relapse; IB: incisional biopsy;

**Clinical-pathological features and PRAME results (% of positive cells, intensity of PRAME stain, and comparison of the three tested scores) of the case series.**
